# Cloning and expression of feline colony stimulating factor receptor (CSF-1R) and analysis of the species specificity of stimulation by colony stimulating factor-1 (CSF-1) and interleukin-34 (IL-34)

**DOI:** 10.1016/j.cyto.2012.11.014

**Published:** 2013-02

**Authors:** Deborah J. Gow, Valerie Garceau, Clare Pridans, Adam G. Gow, Kerry E. Simpson, Danielle Gunn-Moore, David A. Hume

**Affiliations:** The Roslin Institute and Royal (Dick) School of Veterinary Studies, University of Edinburgh, Easter Bush, Midlothian EH25 9RG Scotland, UK

**Keywords:** Macrophage, Ba/F3, Bone marrow, Species specificity, Renal

## Abstract

Colony stimulating factor (CSF-1) and its receptor, CSF-1R, have been previously well studied in humans and rodents to dissect the role they play in development of cells of the mononuclear phagocyte system. A second ligand for the CSF-1R, IL-34 has been described in several species. In this study, we have cloned and expressed the feline CSF-1R and examined the responsiveness to CSF-1 and IL-34 from a range of species. The results indicate that pig and human CSF-1 and human IL-34 are equally effective in cats, where both mouse CSF-1 and IL-34 are significantly less active. Recombinant human CSF-1 can be used to generate populations of feline bone marrow and monocyte derived macrophages that can be used to further dissect macrophage-specific gene expression in this species, and to compare it to data derived from mouse, human and pig. These results set the scene for therapeutic use of CSF-1 and IL-34 in cats.

## Highlights

► Full-length feline CSF-1R has been cloned and expressed in a stable cell line. ► The biological activity of human, mouse and porcine CSF-1 has been evaluated. ► Human and mouse IL-34 are also biologically active on the feline CSF-1R. ► The potential therapeutic applications of CSF-1 is discussed.

## 1 Introduction

Macrophage colony-stimulating factor (CSF-1) controls the proliferation and differentiation of cells of the mononuclear phagocyte lineage [1–3]. The receptor for CSF-1 (CSF-1R, MCSFR, CD115 or FMS) is expressed in all cells of the mononuclear phagocyte lineage including progenitor cells, osteoclasts and dendritic cells [4]. CSF-1R is the proto-oncogene form of the transforming gene of Feline McDonough Sarcoma (SM-FeSV), hence its name FMS [5]. A second ligand for the CSF-1R was first described in humans [6] and subsequent studies confirmed that the two-ligand, one-receptor, system is conserved in birds [7]. Binding of either ligand to the CSF-1R produces receptor dimerization, auto-phosphorylation, activation of down-stream signalling (ERK1/2, Akt) and expression of genes involved in survival and proliferation of the mononuclear phagocyte lineage cells [6,8–10].

Human CSF-1 cDNA was cloned and expressed in the 1980s, and when injected into mice, it promoted an increase in blood monocyte and tissue macrophage numbers [11]. Human CSF-1 is almost as effective as mouse CSF-1 in stimulating mouse macrophage proliferation *in vitro* [12,13] and indeed is equally active on all mammalian species tested (mouse, cat, sheep, dog, and pig). Conversely, mouse CSF-1 bioactivity is restricted to non-primate species [2,7,14–18]. IL-34 has even more restricted species cross-reactivity; with the human and mouse ligands much less active on the other species, although both were active on the pig CSF-1R [2]. CSF-1 has been widely-used to drive proliferation and differentiation of mature macrophages from bone marrow or blood monocyte progenitors in multiple species. In general, CSF-1 stimulated cells are driven towards a more immunosuppressive function [1,19], where another colony-stimulating factor, GM-CSF or CSF-2, drives differentiation of phagocytic cells with antigen-presenting function [20,21]. GM-CSF has been used to produce antigen presenting cells (APCs) from feline bone marrow [22–24], but there are no reports of using CSF-1 to generate feline monocyte derived macrophages.

Recombinant colony stimulating factors and/or antibodies directed against the ligand or the receptor have been tested in a range of animal models and human patients [1,25–27]. Macrophages play an important role in tissue repair in a number of tissues including kidney, liver, heart, brain and lung [28–32] and CSF-1 administration has been shown to promote regeneration in a number of models. For example, in ischaemia reperfusion in mice, a model of acute renal injury, recombinant human CSF-1 administration was able to stimulate macrophage infiltration to promote epithelial repair and to prevent interstitial fibrosis [33].

Acute renal failure in cats can arise from numerous causes [34–40] and has a very poor prognosis [36,41]. Many cats who survive to discharge are azotaemic and, as a result, are likely to have high morbidity and greatly-reduced lifespan, while a second “wave” of apoptosis seems to occur in the recovery phase which may limit further regeneration [42]. The studies in the mouse model suggest that CSF-1 could have a therapeutic benefit in cats with acute renal injury. For this purpose, we need to know whether it would be necessary to produce feline-specific agents. In the present study, we have produced a cat CSF-1R-expressing factor-dependent cell line and evaluated responsiveness of the cat receptor to CSF-1 and IL-34 from multiple species.

## 2 Materials and methods

### 2.1 Cell culture and reagents

The Ba/F3 cell line, transfected Ba/F3 cells and primary bone marrow cells were cultured in RPMI 1640 medium (Sigma) containing 10% HI-FCS, 2 mM L-glutamine, 100 μg/ml streptomycin, and 100 Units/ml penicillin. Untransfected Ba/F3 cells were maintained in medium containing 10% IL-3 from X63 Ag8-653 myeloma cells carrying an expression vector for IL-3 [43,44]. Unless otherwise stated, transfected Ba/F3 cells were maintained in medium containing 10^4^ Units/ml rhCSF-1 (a gift from Chiron Corp., Emeryville, CA, USA). Both Ba/F3 cells and primary bone marrow cells (BMCs) were incubated at 37 °C with 5% CO_2_.

### 2.2 Total RNA extraction and cDNA synthesis

With owner’s consent, tissues were collected from a 5-year-old male Siamese cat that was being euthanized for medical reasons. Tissues were placed immediately in RNA Later (QIAGEN) and stored at room temperature for 24 h until RNA extraction was performed. RNA was extracted from skin, testes, sub-mandibular lymph node, uterus and spleen. Total RNA was prepared using an RNeasy kit (QIAGEN) according to the manufacturer’s instructions, including a DNase digestion step. Feline-specific cDNA was produced using 1 μg of total RNA and reversed transcribed using ImProm-II (Promega). Successful cDNA production without genomic DNA contamination was demonstrated using feline HPRT primers (Table 1) [45].

### 2.3 Expression cloning of feline CSF-1R

Feline PCR primer pairs (Table 1) were designed for amplification of full-length CSF-1R from the incomplete published feline CSF-1R cDNA sequence (Ensembl ENSFCAP00000003348). Amplification was achieved using feline cDNA and expand high-fidelity enzyme (Roche) with 3 mM MgCl_2_ using an initial cycle of 94 °C for 3 min followed by 35 cycles of 94 °C for 30 s, 60 °C for 30 s 72 °C for 3 min and one cycle of 72 °C for 10 min. PCR products were gel purified using a QIAquick gel extraction kit (QIAGEN) and cloned in frame with V5-His C-terminal tag of pEF6/V5-His expression construct using TOPO cloning kit (Invitrogen). DNA sequencing was performed by DNA Sequencing and Services (MRCPPU, College of Life Sciences, University of Dundee, Scotland, www.dnaseq.co.uk) using Applied Biosystems Big-Dye Ver. 3.1 chemistry on an Applied Biosystems model 3730 automated capillary DNA sequence.

### 2.4 Generation of stable cell lines

For generation of stable Ba/F3 cells expressing feline CSF-1R, 5 × 10^6^ Ba/F3 cells were electroporated (300 V, 975 μF) with 10 μg DNA (pEF6_fCSF-1R or empty pEF6 DNA), and selected with 30 μg/ml blasticidin (Invitrogen) and 10% IL-3 for 6 days prior to further selection with 30 μg/ml blasticidin and 10^4^ Units/ml of rhCSF-1.

### 2.5 Immunoblotting

Whole-cell lysate was prepared by lysing 0.5 × 10^6^ cells in 10 mM Tris containing 2% SDS and boiling for 10 min at 100 °C. Protein concentration was determined using DC protein assay (Bio-Rad) with 10 μg of protein mixed with Laemmli buffer (Invitrogen) and 5 mM DTT. Samples were run on a 4–12% gradient precast SDS–PAGE gel (Bio-Rad) and transferred onto polyvinylidene difluoride membrane, as per manufacturer’s directions (Bio-Rad). The membrane was blocked with 5% skimmed milk powder in TBS-Tween 20 at 4 °C overnight prior to being washed and probed with 1:5000 dilution of mouse anti-v5 tag antibody (AbD Serotec MCA1360G) and 1:5000 dilution of anti-mouse IgG HRP conjugated antibody (Cell Signalling Technology, 7076) and detected using enhanced chemiluminescence (ECL) reagents (Amersham, GE Healthcare, UK).

### 2.6 Isolation of feline peripheral blood mononuclear (PBMC) and bone marrow cells (BMC)

A 6-year-old male neutered domestic short-haired cat was euthanized by pentobarbitone for medical reasons and owner’s consent obtained to collect 20 ml of blood and one femur. The femur was dissected, placed in a zip-lock bag and placed on ice. 20 ml of blood was collected into a syringe containing Acid-citrate-dextroseat 1:10 dilution and placed on ice following collection. For BMC collection, both the proximal and distal ends of the femur were removed and using an 18 g needle, cells were flushed with 10 ml RPMI (Sigma) containing 5 mM EDTA to prevent clotting. Cells were washed and re-suspended in red cell lysis buffer (Bio Legend, San Diego, CA) for 5 min, followed by a further 2 washes in PBS. Feline PBMC were isolated using Lymphoprep (Axis-Shield, Oslo, Norway) following manufacturer’s instructions, including the addition of red cell lysis buffer as above.

### 2.7 Feline peripheral PBMC and BMC stimulation with recombinant human CSF-1

Ten million PBMC and BMC were cultured in 60 mm bacteriological plates with 4 ml RPMI supplemented with 10^4^ Units/ml rhCSF-1, and incubated for 8 days at 37 °C, 5% CO_2_.

### 2.8 Preparation of cells for cytospin

After 8 days in culture, adherent cells from both blood and BMC cultures were recovered by repeated flushing of medium over the bottom of the culture dish until all adherent cells were removed. Cells were counted and 0.5 × 10^6^ cell/ml were collected with 100 μl of cell suspension placed into the cytospin chamber (Thermo) and centrifuged at 300 rpm for 3 min. Slides were air-dried and fixed in 100% methanol for 5 min prior to staining with Leishman’s stain (Sigma L6254) for the identification of macrophage nuclei. Cytospins were examined under 20× magnification for cellular morphology.

### 2.9 Phagocytosis assay

Following harvest of day 8 adherent cells from both blood and BMC cultures (as above), 1 × 10^6^ cells/ml were plated/well of a 6-well plate in duplicate and cultured overnight at 37 °C, 5% CO_2._ Phagocytosis was initiated by the addition of FITC conjugated Zymosan bio-particles (Molecular Probes) at a particle:cell ratio of 10:1, followed by further incubation for 1 h. Phagocytosis was stopped by the addition of 500 μl/well of ice-cold PBS, followed by 2 PBS washes. Cells were analysed for Zymosan particle uptake using fluorescence microscopy (Zeiss LSM710).

### 2.10 Cell viability assays

Stable Ba/F3 cells expressing feline CSF-1R were maintained in culture with RPMI supplemented with 10^4^ Units/ml rhCSF-1 prior to MTT assay. 2 × 10^4^ cells/well were plated in quadruplicate and appropriate treatment (serial dilutions of rhCSF-1, rpCSF-1, rmCSF-1, (R&D Systems 416-ML), rhIL-34 (R&D Systems 5265), or rmIL-34 (R&D Systems 5195) added to make a total volume of 100 μl per well. Cells were incubated for 48 h at 37 °C, 5% CO_2_, after which 10 μl of MTT (Sigma–Aldrich M5655) was added directly to each well (final concentration of 0.5 mg/ml) and incubated at 37 °C for 3 h prior to solubilisation with 100 μl of solubilisation agent (0.1 M HCl, 10% Triton x-100 and isopropanol) and overnight incubation. Plates were read at 570 nm with reference wavelength of 405 nm.

### 2.11 3D modelling of contact amino acids

3D models in PDB format were generated with 3D-Jigsaw (http://bmmcancerresearchuk.org/3djigsaw/) using structure-based alignments (performed by Domain Fishing). 3D models of human CSF-1R (PDB 4DKD) and mouse CSF-1R (PDB 4EXP), were obtained and viewed in FirstGlance in Jmol (http://firstglance.jmol.org). The 3D model of feline CSF-1R was generated using 3D-Jigsaw with the mouse CSF-1R structure as template (3EJJ). Non-conserved contact amino acids for IL-34 and CSF-1 binding of the CSF-1R were identified using recently-published data [46–48] and highlighted.

## 3 Results

### 3.1 Cloning of feline CSF-1R

**Fig. 1:**
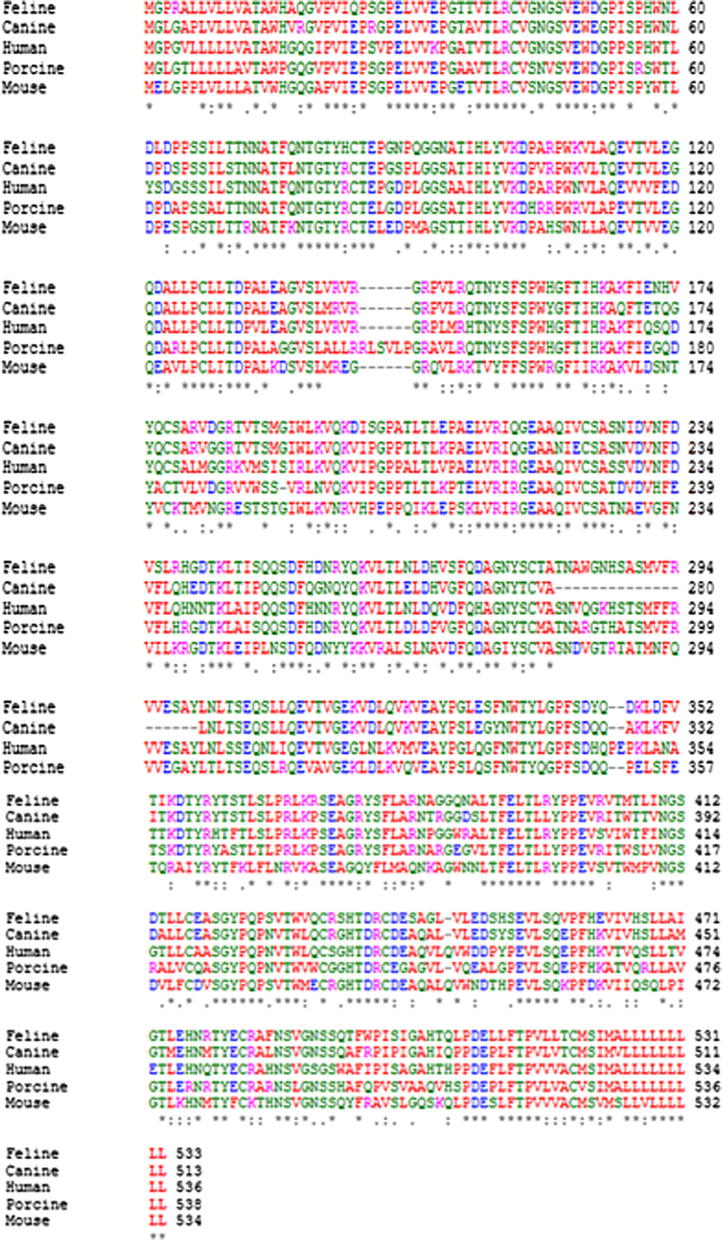
Alignment of cloned feline CSF-1R extracellular domain with human, pig and mouse. Alignment was performed using Clustal W (http://www.ebi.ac.uk/Tools/msa/clustalw2/). The alignment clearly demonstrates the high level of homology that exists in the CSF-1R extracellular domain containing the IL-34 and CSF-1 binding sites. Of particular note is the high level of homology between both the feline and canine CSF-1R (88%) and feline and human CSF-1R (83%) while the homology between feline and the mouse and pig CSF-1R share 75%and 80% homology respectively. The red colour represents small hydrophobic amino acids, blue colour represents acidic amino acids, magenta denotes basic amino acids, and the green colour corresponds to hydrophilic or polar amino acids. Identical amino acids are represented by “∗”, conserved substitutions are represented by “:” and semi-conserved substitutions are represented by “.”.

Woolford et al. [14] previously cloned feline CSF-1R cDNA from splenic cDNA template but we identified a greater abundance of CSF-1R cDNA using the lymph node template. Agarose gel electrophoresis of the PCR products revealed the expected single band of approximately 3000 base pairs in this tissue. Following gel purification, feline CSF-1R was successfully cloned in frame with V5-His C-terminal tag of pEF6 V5-His TOPO plasmid. The cDNA and protein sequences were confirmed and multiple species alignments of CSF-1R performed (Fig. 1). The feline CSF-1R extracellular domain shares 88% homology with the canine CSF-1R, 83% homology with human CSF-1R, and 75% with the mouse CSF-1R, while feline and pig share 80% homology. The cloned feline CSF-1R encodes the full-length receptor of 982 amino acids, including a 19 amino acid signal peptide (Met1 – Gly19) and a 963 amino acid mature chain (Val20 to Cys982). The sequence is identical to the previously-published feline CSF-1R [14]. The earlier study emphasised variation between feline *c-fms* and the transforming genes of the feline sarcoma virus, *v-fms*.

**Fig. 2:**
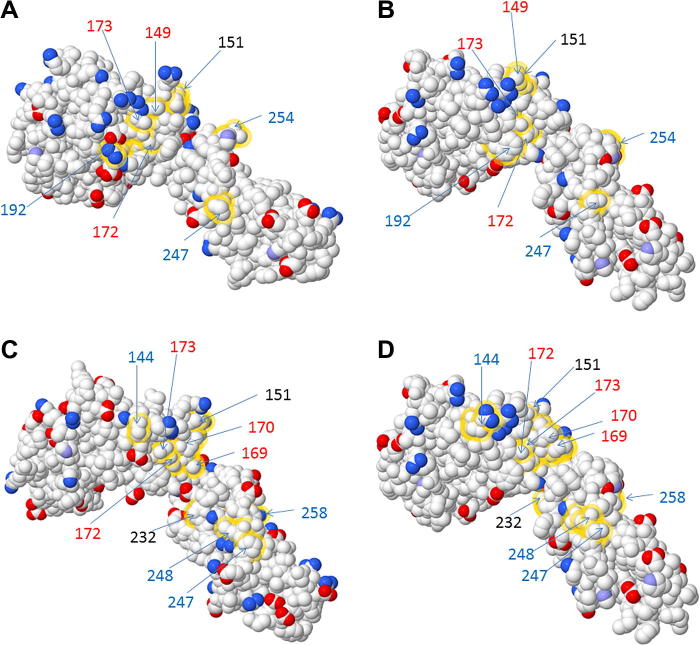
3D models of non-conserved CSF-1 and IL-34 contact amino acids between human, mouse and cat CSF-1R. 3D models demonstrating the charged amino acid changes between human (A) and cat (B) CSF-1R and between mouse (C) and cat (D) CSF-1R. Models were generated using the PDB files for mouse CSF-1R (3EJJ) and human CSF-1R (4DKD chain X). The mouse PDB file (3EJJ) was used as a template to generate the cat CSF-1R. Published contact amino acids for both human and mouse CSF-1R binding sites for CSF-1 and IL-34 were analysed and non-conserved contact amino acids highlighted using FirstGlance (http://firstglance.jmol.org). Non-conserved amino acids for CSF-1 binding are represented by black numbers; red numbers indicate CSF-1 and IL-34 binding, while blue numbers indicates IL-34 binding. Positively charged atoms are represented by blue colour and negatively charged atoms by red colour. Medium blue coloured atoms denote partially charged atoms.

The availability of the crystal structure for the human CSF-1/IL-34 complexes with CSF-1R permits a structure-based modelling of the same complexes in other species. The contact residues for the ligands are not conserved across species. The human and feline CSF-1R binding sites for CSF-1 and IL-34 differ by 7 non-conserved amino acids (Fig. 2A and B). Similarly, there are 10 contact amino acid differences between mouse and feline CSF-1R for the CSF-1 and IL-34 binding sites (Fig. 2C and D). As noted previously in an analysis of pig CSF-1R [2], it is difficult to predict from these changes whether the cat receptor will bind CSF-1 or IL-34 from other species, and the results may inform both therapeutic applications and structure–function predictions.

### 3.2 Production of feline CSF-1R expressing cells

**Fig. 3:**
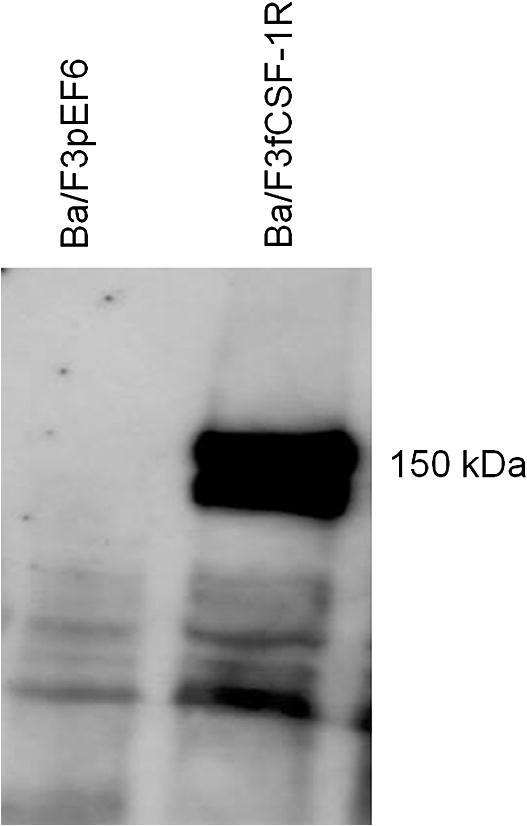
Western blot of transfected Ba/F3 cells expressing feline CSF-1R. A western blot was performed on Ba/F3 cells expressing feline CSF-1R. Cells were cultured in rhCSF-1 prior to collection of cell lysate. Cell lysate was collected from both Ba/F3 cells transfected with feline CSF-1R, but also Ba/F3 cell transfected with empty pEF6 vector as a negative control. A band at 150 kDa, corresponding to the predicted size is clearly visible for the feline CSF-1R, with no band identified for the negative control.

To enable studies of the cat CSF-1R binding specificity, we stably transfected the IL-3 dependent Ba/F3 cell line as previously described for the human, chicken and pig receptors with full-length feline CSF-1R [2,7,49]. Stable clones were initially selected for their survival in blasticidin, followed by further selection in rhCSF-1. The presence of feline CSF-1R in Ba/F3 cells (Ba/F3fCSF-1R) permitted proliferation in response to rhCSF-1 and removed the dependence of these cells for IL-3. Western blot analysis of these cells demonstrated detectable expression of feline CSF-1R at a similar level to porcine CSF-1R expressed in Ba/F3 cells [2] (Fig. 3).

### 3.3 Activation of feline CSF-1R with human, mouse and porcine CSF-1

**Fig. 4:**
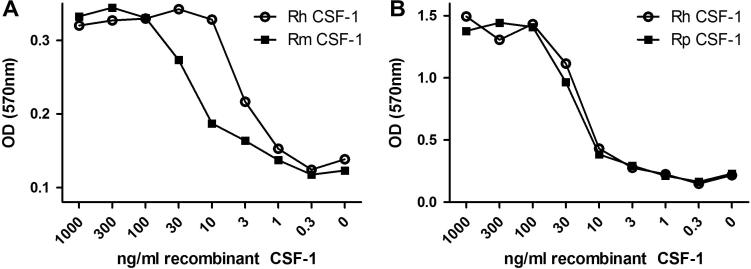
Activity of human, mouse and porcine recombinant CSF-1 on feline CSF-1R expressed in Ba/F3 cells. An MTT cell viability assay was used to assess the biological activity of recombinant human, mouse and porcine CSF-1 in the feline CSF-1R. (A) Both human and mouse CSF-1 are biologically active on the feline CSF-1R, with mouse CSF-1 demonstrating reduced activity compared to human CSF-1. (B) Comparing the effects of human and porcine CSF-1 on the feline CSF-1R demonstrates that these recombinant proteins are identical in their activation.

The MTT bioassay for assessing the response of transfected Ba/F3 cells to CSF-1 has been previously described and optimised [2]. Both the parent Ba/F3 and Ba/F3 cells expressing feline CSF-1R survived and proliferated in IL-3. Human, mouse and porcine recombinant CSF-1 allowed the Ba/F3 cells expressing feline CSF-1R to survive, but with distinct efficacy. The actual EC_50_ differs between experiments. Because the cells consume the factor, the assay is sensitive to precise cell number and duration. In a side-by-side comparison, mouse CSF-1 was substantially less active than human CSF-1 (Fig. 4A) whereas human and pig CSF-1 demonstrated virtually identical activity on the feline CSF-1R (Fig. 4B), comparable to their reported activity on the pig CSF-1R [2].

### 3.4 Cultivation of feline bone marrow and peripheral blood mononuclear cells with human CSF-1

**Fig. 5:**
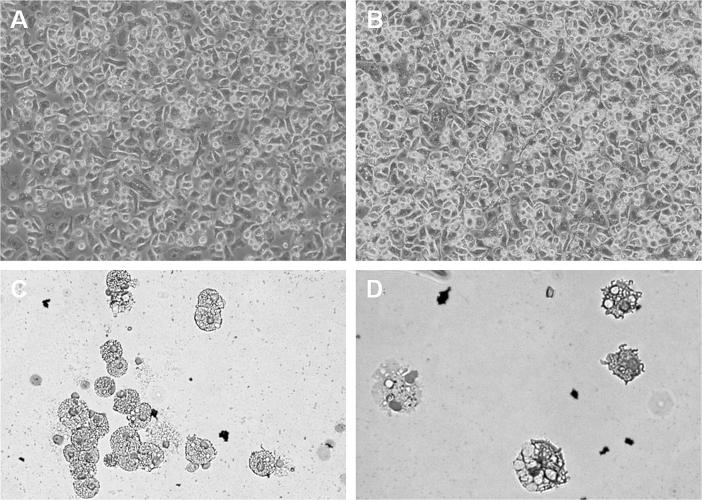
Feline monocyte derived and bone marrow derived macrophages. Feline peripheral blood monocytes and bone marrow cells progenitor cells were cultured with 10^4 ^Units/ml CSF-1 for 8 days. For both the peripheral blood mononuclear cell (A), and bone marrow cell (B), cultures with rh-CSF-1, cells were adherent, had increased in size and granularity and divided, thus producing blood monocyte and bone marrow derived macrophages respectively. Cytospin analysis of the blood monocyte derived macrophages (C) and bone marrow derived macrophages (D) demonstrate that both populations display macrophage-like morphology.

**Fig. 6:**
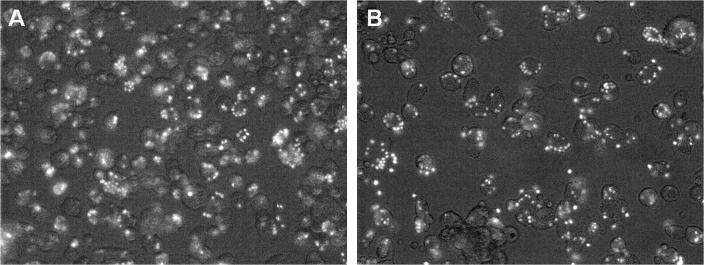
Phagocytic activity of feline monocyte and bone marrow derived macrophages. Cells were cultured for 10 days in rh-CSF-1, prior to the addition of FITC-labelled Zymosan particles for 1 h. Both cell populations were highly phagocytic and ingested numerous particles of Zymosan after the 1 h incubation. (A) BMC and (B) PBMC.

We have previously reported the production of bone marrow-derived macrophages from pig marrow using human CSF-1 [50]. Similar to that study’s findings, feline BMC and PBMC cultivated in recombinant human CSF-1 increased in number, granularity, and size, and became adherent over an 8 day period, demonstrating clear macrophage-like morphology (Fig. 5A and B). Cells not cultured with CSF-1 did not adhere, grow or survive. This was particularly evident for the bone marrow-derived-macrophages (Fig. 5C and D). Both macrophage populations of primary cell populations were potently phagocytic and ingested particles after one hour of incubation (Fig. 6). This result demonstrates that feline CSF-1R in its native context is able to bind and respond to recombinant human CSF-1. By analogy to our previous study on the pig, this method would allow the freezing of marrow progenitors from euthanized cats for multiple future studies *in vitro*.

### 3.5 Activation of feline CSF-1R with IL-34

**Fig. 7:**
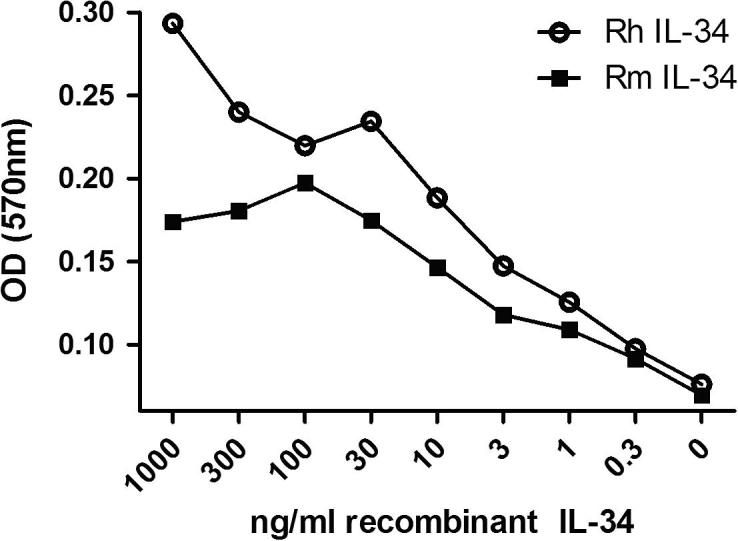
Activity of recombinant human and mouse IL-34 on feline CSF-1R expressed in Ba/F3 cells. An MTT cell viability was used to assess the biological activity of human and mouse IL-34 on expressed feline CSF-1R. Both human and mouse IL-34 are biologically active on the feline CSF-1R in a dose dependant manner. The EC_50_ for mouse IL-34 on the feline CSF-1R was approximately 3-fold higher than for human IL-34.

**Fig. 8:**
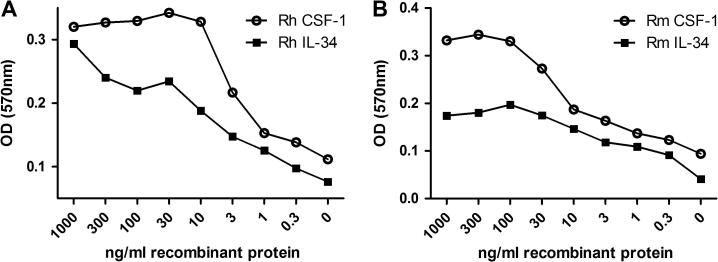
Comparing the activity of human and mouse IL-34 and CSF-1 on expressed feline CSF-1R. An MTT cell viability assay was used to compare the biological activity of human and mouse IL-34 with human and mouse CSF-1. For both human (A) and mouse (B), IL-34 has demonstrated approximately half the biological activity compared to human and mouse CSF-1.

Human and mouse IL-34 have similar activity on the pig CSF-1R [2]. Using the MTT bioassay with Ba/F3 cells expressing feline CSF-1R, both human and mouse IL-34 were able to bind and activate the feline CSF-1R, in a dose-dependent manner (Fig. 7), but the EC_50_ for mouse IL-34 on the feline CSF-1R was approximately threefold higher than for human IL-34. Neither human, nor mouse recombinant IL-34 was as active as human CSF-1 under the same conditions (Fig. 8).

## 4 Discussion

We confirmed the published cDNA and protein sequence of feline CSF-1R and stably cloned the cat receptor into the Ba/F3 cell line. Activation of feline CSF-1R by recombinant human CSF-1 was previously tested by Woolford et al. [14]. Expression of oncogenic forms of feline CSF-1R in Rat-2 cells, which ordinarily are unable to grow in soft agar [51], permitted the formation of colonies in soft agar upon the addition of human CSF-1 [14]. However, the native receptor was insufficient to cause transformation even in the presence of human CSF-1. This study also gave no insight into relative efficacy. In the current study, the addition of either human or mouse CSF-1 to Ba/F3 cells expressing full length feline CSF-1R produced survival and proliferation of cells that are dependent on CSF-1 for survival. Mouse CSF-1 was substantially less active on the feline receptor than human CSF-1. In this respect, the cat CSF-1R is idiosyncratic compared to the other species tested. Mouse and pig CSF-1R respond equally to mouse, pig and human CSF-1, human CSF-1R responds to human and pig, but not to mouse CSF-1 [2].

Publication of the mouse CSF-1: CSF-1R contact amino acids [48] permits a structure-based analysis of receptor specificity. There are 6 non-conserved contact amino acids (His6, Asn13, Phe55, Glu78, Arg79, and Asn85 in mouse) between mouse and human CSF-1 [2]. His6 is conserved in cat, and is Asn in pigs, but is the bulky aromatic amino acid, Tyr, in humans. This difference could explain why mouse CSF-1 can bind the pig and cat CSF-1R, but not the human receptor. Amino acids 78 and 79 of CSF-1 are Glu and Arg (positive) in mice, Val and Thr in cat (neutral), and Val and Gln (neutral) in both human and porcine CSF-1. These differences could explain the relatively lower efficacy of mouse CSF-1 on the feline CSF-1R.

Both human and mouse IL-34 were also able to activate the feline CSF-1R *in vitro*; again the mouse ligand was less active. IL-34 is more species-specific than CSF-1 as identified by the reduced activity of human IL-34 on the mouse CSF-1R [8]. Human IL-34 and CSF-1 were similarly active on the pig CSF-1R [2] but with the cat receptor the EC_50_ for human IL-34 was substantially higher than for CSF-1. Again, the differences between the species probably reflect differences in CSF-1R contact amino acids for IL-34 that may account for the reduced activity of mouse IL-34 on the cat CSF-1R. The difference that is most likely to disrupt function is Gln258 (human and cat) which is substituted with a charged Lys in the mouse CSF-1R.

The growth of macrophages from bone marrow cells using recombinant CSF-1 has been described previously for human, mouse, chicken and pig bone marrow cells [7,17,50,52–54]. Culture of feline macrophages is a valuable tool to allow the *in vitro* study of the effects of drugs, e.g. chemotherapeutic agents, which may cause immune-suppression or immune-modulation in cats. Equally, the mechanisms of feline infectious diseases that can infect macrophages, e.g. FIV or mycobacteria, may be further investigated. It will be of interest to compare the response of feline BMDMs to LPS in a similar fashion to the analysis performed for mice, human and porcine BMDMs [50,55].

We have demonstrated previously that both recombinant human and porcine CSF-1 proteins can activate feline and canine CSF-1Rs using bone marrow aspirates [2]. The primary marrow cells used in the present study were harvested post-mortem, by repeated flushing from the femur. An earlier study Daniel et al. [56] noted that feline primary bone marrow cultures could be successfully maintained without the addition of exogenous CSF-1 due to the production of CSF-1 by the adherent bone-marrow cell population. This is a rather less-reproducible and inefficient approach. Bone-marrow cells can be frozen and thawed, so that cells preserved from a small number of animals can provide a long term resource for studies of macrophage biology.

Recent studies investigating the effects of mesenchymal stem cell (MSC) therapy in rodent models including chronic renal failure and glomerulonephritis have demonstrated that MSC therapy can result in beneficial effects [57,58]. In cats, autologous intra-renal injections of either adipose tissue-derived or bone-marrow-derived mesenchymal stem cells demonstrated modest improvements in glomerular filtration rate (GFR) and serum biochemical markers of renal disease [59]. MSCs are highly proliferative, undifferentiated cells that can self-renew [60,61]. Due to these properties, MSCs are being suggested as therapeutic options for a range of diseases including chronic renal failure in cats [59,62]. CSF-1 mRNA and protein are constitutively expressed [63–65] and, given the trophic functions, would be candidate mediators of some of the effects of MSC. Previous studies using non-species-specific colony stimulating factors for therapy have been hampered by the development of auto-antibodies. For example, the administration of rhGM-CSF to healthy dogs has been reported to produce neutralising antibodies after 10–12 days [66]. Similarly, the administration of rhGM-CSF to cats with FIV triggered neutralising antibodies in 75% of the cats, 35 days after a 2-week treatment protocol [67]. A practical therapy for cats based upon CSF-1R agonist is likely to involve the production of the species-specific protein, but this is costly. For therapeutic trials, our data shows that human and pig CSF-1 or IL-34 would have similar efficacy and could be considered for potential therapy. For acute therapy, the generation of neutralising antibodies may not be a significant issue.

In conclusion, we have developed an *in vitro* system for the study of feline macrophages which will allow further investigation of macrophage related diseases and the effects of therapy on these cells. The feline CSF-1R has been cloned and expressed in Ba/F3 cells and used to assess the activity of non-species-specific CSF-1 and IL-34. This assay also offers the possibility of screening for antagonists, including blocking antibodies, which might have applications in inflammatory disease and malignancy [1].

## Figures and Tables

**Table 1:** Table of primers used for production of feline cDNA and cloning of feline CSF-1R. A kozak sequence (highlighted in bold) was included in CSF-1R forward primer for optimal translation initiation. Feline HRPT primers from Penning et al. [45].

Primer name	Primer sequence 5′–3′	Tm (°C)	Size (bp)
Feline HPRT Forward	ACTGTAATGACCAGTCAACAGGGG	60.0	210
Feline HPRT Reverse	TGTATCCAACACTTCGAGGAGTCC	60.0	–
Feline CSF-1R Forward	**GCC** ATG GGC CCA AGG GCT	61.5	2946
Feline CSF-1R Reverse	GCA GAA CTG GTA GTT GTT GGG CTG	61.9	–
